# Alleviation of tannery wastewater toxicity in *Spinacia oleracea* through application of metal oxide nanoparticles

**DOI:** 10.1038/s41598-025-34464-8

**Published:** 2026-02-28

**Authors:** Ihsan Elahi Zaheer, Shafiq ur Rehman, Mehwish Liaquat, Maryam Saif, Fareeha Kanval, Sarvet Jehan, Shafaqat Ali, Abdulrahman Alasmari, Sarah Owdah Alomrani, Ibrahim Al-Ashkar, Ayman El Sabagh, Abdul Ghafoor

**Affiliations:** 1 Department of Environmental Sciences, University of Okara, Okara, 56300 Pakistan; 2 Department of Botany, University of Okara, Okara, 56300 Pakistan; 3 Department of Horticulture, PMAS Arid Agriculture University, Rawalpindi, Pakistan; 4 Institute of Soil and Environmental Sciences, PMAS Arid Agriculture University, Rawalpindi, Pakistan; 5 Department of Environmental Sciences, Government College University, Faisalabad, 38000 Pakistan; 6 Department of Biological Sciences and Technology, China Medical University, Taichung, 40402 Taiwan; 7 Department of Biology, Faculty of Science, University of Tabuk, 71491 Tabuk, Saudi Arabia; 8 Biodiversity Genomics Unit, Faculty of Science, University of Tabuk, 71491 Tabuk, Saudi Arabia; 9 Department of Biology, College of Science and Arts, Najran University, 66252 Najran, Saudi Arabia; 10 Department of Plant Production, College of Food and Agriculture Sciences, King Saud University, P.O. Box 2460, 11451 Riyadh, Saudi Arabia; 11 Department of Field Crops, Faculty of Agriculture, Siirt University, Siirt, Turkey; 12 Centre for Water and Environmental Studies, King Faisal University, 31982 Al-Ahsa, Saudi Arabia

**Keywords:** Chromium stress, *Spinacia oleracea*, Nanotechnology, Oxidative stress, Antioxidant enzymes, Biochemistry, Environmental sciences, Plant sciences

## Abstract

Hexavalent chromium [Cr(VI)] rich tannery wastewater has negative impacts on physiological functions and growth of plants. We examined the effectiveness of foliar application of metal oxide nanoparticles including magnesium oxide, zinc oxide and iron oxide in mitigating chromium induced toxicity in *Spinacia oleracea* under 0%, 50%, and 100% tannery wastewater irrigation levels. The results reveal that chromium stress notably reduced plant growth, chlorophyll content, increased oxidative stress (H_2_O_2_ and MDA), electrolyte leakage and build up of Cr both in roots and shoots of plants. The stress also reduced mineral nutrient acquisition including Mg, Fe and Zn. However, foliar application of MgO, FeO and ZnO nanoparticles strengthened the activities of antioxidant enzymes (SOD, POD and CAT) thus resulting in lowered oxidative stress and concentration of Cr within plant body. Application of nanoparticles helped the plants to absorb more mineral nutrients (Mg, Fe and Zn) as well. ZnO nanoparticles proved to be the most effective in mitigating Cr stress in *Spinacia oleracia* followed by MgO and FeO. This study demonstrates the effectiveness of ZnO NPs along with MgO and FeO in preventing Cr-actuated pressure in degraded conditions.

## Introduction

The rising ecological contamination by heavy metals, primarily driven by industrial activities and human actions, has posed significant challenges. The leather production industry is a major source of heavy metal containing toxic wastewater, exerting a more substantial environmental impact compared to many other industries, both directly and indirectly^1^. Chromium is a common contaminant in tannery effluent^1,2^ and Cr (VI) is a metallic compound which exhibits exceptional mobility, solubility, as well as toxicity^3^. Agricultural lands are increasingly being degraded by the industrial effluent discharge^4^. The metallic substances present in the tannery effluent may accumulate and circulate throughout the environment as well as food chain^5^, affecting the productivity of the ecosystem as a whole^6^. Excess chromium in plants can harm them by increasing oxidative stress^7^, undermine their photosynthesis capacities and growth, and ultimately lower crop produce quality^8^. Besides, Chromium can interfere mineral uptake in vegetation as well^9^.

Leafy vegetables are especially vulnerable to sewage-related risks because of their capacity to amass foreign substances^10^. Throughout Pakistan, spinach, a vegetable rich in nutrients, has been produced to a high standard using conventional water systems^11^. Unique traits of *Spinacia Oleracea*, such as its high development productivity and response to different heavy metals under stressful conditions, have sparked a great deal of research^12,13^. It has been recorded that chromium exposure inhibits the growth of *Spinacia oleracea* by lowering the biomass, compromising physiological processes, and causing oxidative stress^13,14^. The presence of chromium in the environment results in significant reduction in the usual biomass output of spinach^15^.

Innovative wastewater remedial measures are made possible by nanotechnology, which has benefits including decreased energy usage, scalability, and a less adverse environmental impact^16–19^. Using nanoparticles as adsorbents has become an effective method to reduce the phytotoxicity of heavy metals, offering a practical and affordable alternate for existing remediation options^20,21^. Utilizing metal oxide nanoparticles, such as ZnO, FeO, and MgO, has proved to improve growth and minimize oxidative stress in plants^22–24^ as these are also essential plant nutrients. Application of nanoparticles of zinc oxide, a micronutrient^25^, has been reported to promote plant growth and development by lowering oxidative stress and Cr concentration in plant roots^26,27^ thus alleviating Cr toxicity. Similarly, topically applied MgO nanoparticles (MgO-NPs) improved soybean photosynthetic efficiency and growth while reducing oxidative stress and arsenic uptake in arsenic-contaminated environments^28^. Besides, MgO-NPs improve nutrient uptake and heavy metal stress tolerance in a variety of plants^29^, including soybean and brassica^28,30^. Iron oxide nanoparticles (NPs) have potential for Cr stress mitigation in plants as well^31^. They significantly improved plant growth, biomass, and photosynthetic activity by enhancing chlorophyll contents and alleviating oxidative damage under chromium stress^32^.

Several plant species have been researched for the combined application of iron FeO and ZnO nanoparticles. ZnO and FeO NPs decreased heavy metal toxicity in rice (*Oryza sativa*), maize (*Zea mays*), wheat (*Triticum aestivum*), and other edible plants under a variety of stress situations^33^. Zinc oxide and iron oxide nanoparticles seed priming increased wheat development, decreased cadmium (Cd) accumulation, and improved nutrient concentrations in roots, shoots, and grains^24^. Co-application of ZnO and FeO NPs improved growth and decreased chromium uptake in lettuce (*Lactuca sativa*)^34^. The combined effects of iron and zinc oxide nanoparticles on plant health have been studied, however studies examining the combined application of ZnO, FeO, and MgO NPs are notably absent. This gap in the literature offers a great chance for further study to thoroughly assess their combined effectiveness in reducing heavy metal stress and enhancing plant health.

The foremost aim of this study was to examine the impact of Zn, Mg, and Fe NPs on the mitigation of Cr toxicity in spinach. More precisely, the research sought to assess their collective effects on plant growth, biomass accumulation, oxidative stress, and Cr absorption under conditions of Cr contamination.

## Results

### Plant growth attributes

**Fig. 1:**
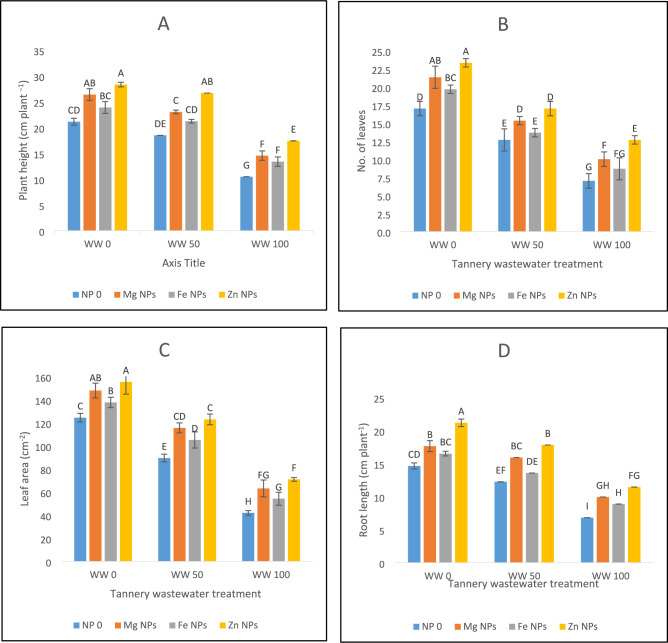

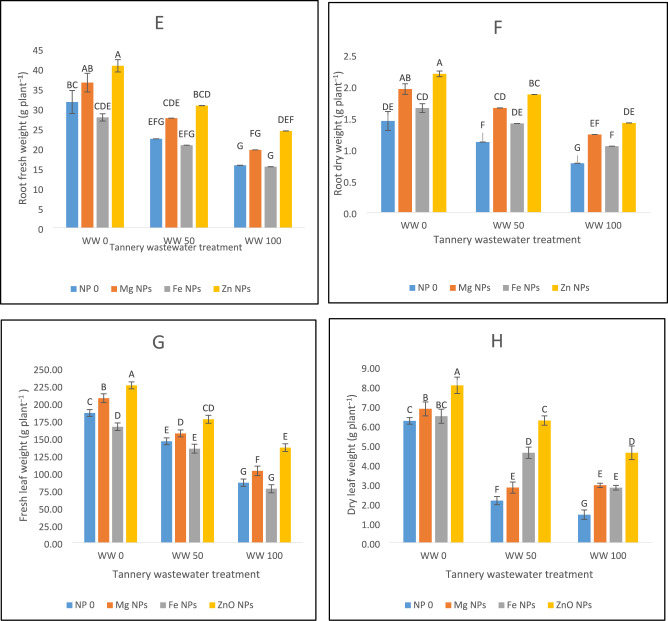
The effects of Fe, Zn and Mg nanoparticles (at concentrations of 25, 50 and 75 mg l^−1^, respectively) on the morphological attributes of spinach plants when irrigated with (0%, 50%, and 100%) wastewater from tannery: (**A**) Plant height, (**B**) Number of leaves on each plant, (**C**) Leaf area, (**D**) Root length, (**E**) Root fresh weight, (**F**) Root dry weight, (**G**) Fresh leaf weight, (**H**) Dry leaf weight. Every treatment has three separate replications. Letters assigned to the values indicate significance at α = 5%.

Figure 1 depicts significant modifications of spinach plant growth attributes when exposed to wastewater irrigation stress. Upon irrigation with tannery wastewater, the morphological growth parameters of spinach plants showed a significant reduction. A reduction of 51% in plant height was observed under 100% wastewater irrigation stress as compared to 12% reduction under 50% wastewater stress (Fig. 1A). Likewise, the number of leaves of spinach experienced a decrease of 56% and 47%, respectively, when subjected to 50% and 100% wastewater stress (Fig. 1B). Leaf area (39% and 67%) and root length (16% and 53%) were significantly reduced under 50% and 100% wastewater levels, accordingly (Fig. 1C,D). A significant reduction of 41% and 65% in root fresh weight, and 18% and 51% in root dry weight were observed, accordingly, under 50% and 100% wastewater stress (Fig. 1E,F). Similarly, under 50% and 100% wastewater stress, dry leaf weight showed a reduction of 32% and 68% whereas fresh leaf weight decreased by 24% and 60%, respectively (Fig. 1G,H). Plants irrigated with 100% water indicated the most significant inhibition of all the characteristics (Fig. 1).

The application of magnesium oxide, iron oxide and zinc oxide nanoparticles aided the spinach plants’ recovery from applied stress of tannery wastewater, resulting in an increase in growth rate and plant biomass. The results (Fig. 1A) of this study showed that the foliar application of 25 mg Fe NPs, 50 mg Zn NPs and 75 mg Mg NPs, on plants irrigated with 0, 50 and 100 percent tannery wastewater improved height of plant. Mg NPs increased height of spinach plant by 29%, 30%, and 29% under control, 50%, and 100% wastewater conditions, respectively. Fe NPs increases plant height by 25%, 27%, and 21% under application of wastewater. Zn proved to be most significant in improving plant height under stress conditions (Fig. 1A).

Application of tannery wastewater resulted in reduced number of leaves by 59% under 100% wastewater stress and 25% under 50% wastewater treatment (Fig. 1B). Administration of Zn NPs increased no. of leaves by 37%, 34% and 81% under 0, 50 and 100 percent wastewater stress, accordingly. Similarly, a rise in no. of leaves by 25%, 21%, and 42% was noted upon Mg NPs usage whereas Fe NPs enhanced no. of leaves by 15%, 7%, and 23%, respectively, under 0, 50 and 100% wastewater stress. Zn NPs proved to be the most effective in increasing the no. of leaves in stress conditions (Fig. 1B). Root length was shortened by 16% under 50% wastewater stress and 53% under 100% wastewater stress against control. Applying Zn NPs exhibited 44% increase in root length in control conditions, and 45% and 68% in 50 and 100% wastewater stress, accordingly. In case of 0, 50% and 100% wastewater irrigation stress, Mg NPs showed an increase in root length by 20%, 30% and 46%, and Fe NPs by 12%, 10% and 30%, respectively (Fig. 1D).

In response to wastewater irrigation, a diminishing of fresh root weight was noticed indicating a 24% reduction in 50% wastewater stress and 56% when plants faced 100% tannery wastewater stress. Zn NPs proved to be most effective in increasing the fresh root weight by 28% in 50% stress conditions and 35% in 100% wastewater stress. Mg increase fresh root weight by 17%, 16% and 26%, in 0, 50 and 100% conditions of wastewater stress (Fig. 1E). Wastewater application caused a major decline in fresh leaf weight which showed a 21% decrease in 50% wastewater stress and 49% when 100% stress was applied as compared to control. Zn proved to be most effective in increasing fresh leaf weight by 23 in normal conditions, 21% in 50% wastewater stress and 60% in 100% wastewater stress. Mg NPs increased leaf weight by 12%, 8%, and 20%, and Fe NPs remained statistically insignificant in same conditions of wastewater stress (Fig. 1G).

### Chlorophyll concentration and attributes of gaseous exchange

**Fig. 2:**
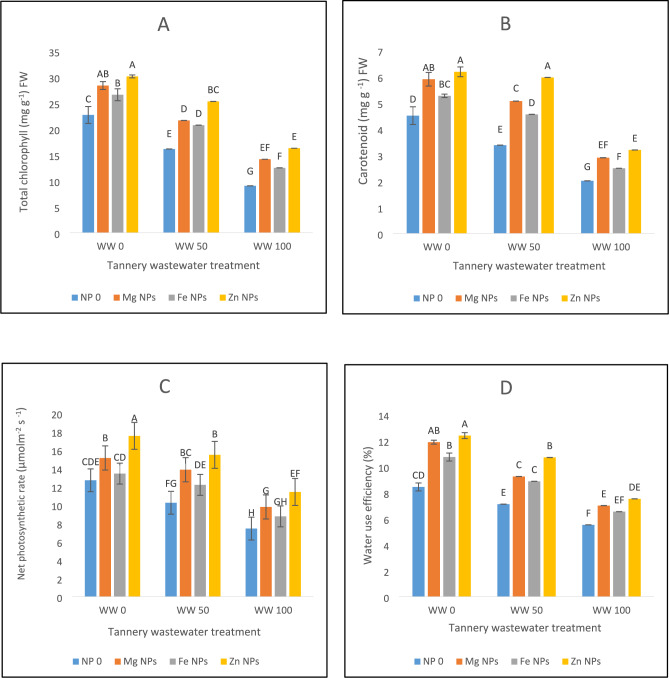

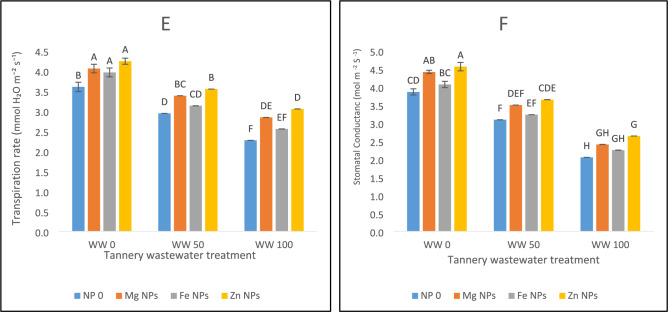
The effects of Fe, Zn and Mg nanoparticles (at concentrations of 25, 50 and 75 mg l^−1^, respectively) on the Chlorophyll contents and gaseous exchange attributes of spinach plants when irrigated with (0%, 50%, and 100%) wastewater from tannery: (**A**) Total chlorophyll contents, (**B**) Carotenoid contents, (**C**) Net photosynthetic rate, (**D**) Water use efficiency, (**E**) Transpiration rate, (**F**) Stomatal conductance. Each treatment has three replicates. Letters assigned to the values indicate significance at α = 5%.

A significant reduction of total chlorophyll contents and hinderances in gaseous exchange was seen in spinach under wastewater irrigation stress conditions as depicted in Fig. 2. Chlorophyll content decreased by 28% in 50% wastewater stress and in 100% wastewater chlorophyll content decreased by 60% (Fig. 2A). Combined application of NPs enhances the chlorophyll content of spinach plants under stress environment. In normal conditions, Zn NPs increased the chlorophyll content by 32%, Mg NPs enhanced the chlorophyll levels by 13% and Fe NPs by 9%. Zn NPs proved to be the most effective in enhancing chlorophyll content under non-stress conditions. In 50 and 100% stress conditions, Zn NPs increase proved to be the most effective in increasing chlorophyll content (Fig. 2A). In wastewater stress, carotenoid concentration also reduced significantly. Carotenoid levels decreased by 25% in 50% wastewater stress and 55% in 100 percent wastewater as compared to control. Zn NPs increase the carotenoid content by 37% under normal conditions. Mg NPs and Fe NPs increase the carotenoid level by 17% and 11%. At 100% stress, Zn NPs increases the carotenoid content by 59% (Fig. 2B).

During wastewater stress, spinach’s net photosynthetic rate decreased. Photosynthesis declined 19% under 50% wastewater stress and 41% under 100% stress. Under normal conditions, Zn NPs elevated the photosynthetic rate by 38%, the largest improvement. The photosynthetic rate was increased by 19% and 24% by Mg and Fe NPs, respectively. Zn NPs improved photosynthesis by 51% at 50% stress level (Fig. 2C). In spinach, wastewater stress has a detrimental effect on water use efficiency (WUE). WUE decreased by 15% and 34%, respectively, at 50% and 100% wastewater stress. Under normal conditions, Zn NPs significantly increase WUE by 47%, Mg and Fe NPs increase WUE by 24% and 18%, respectively. Zn NPs produced the best recovery in water use efficiency, increasing WUE by 50% at 50% stress and 36% at 100% stress (Fig. 2D). Wastewater stress significantly reduced the transpiration rate and stomatal conductance in spinach (Fig. 2E,F). Under both the normal and stress conditions, application of Zn, Mg and Fe oxide NPs improved stomatal conductance and transpiration rate. Zn NPs showed the greatest improvement followed by Mg and Fe NPs.

### Electrolyte leakage and oxidative stress markers (H_2_O_2_, MDA)

**Fig. 3:**
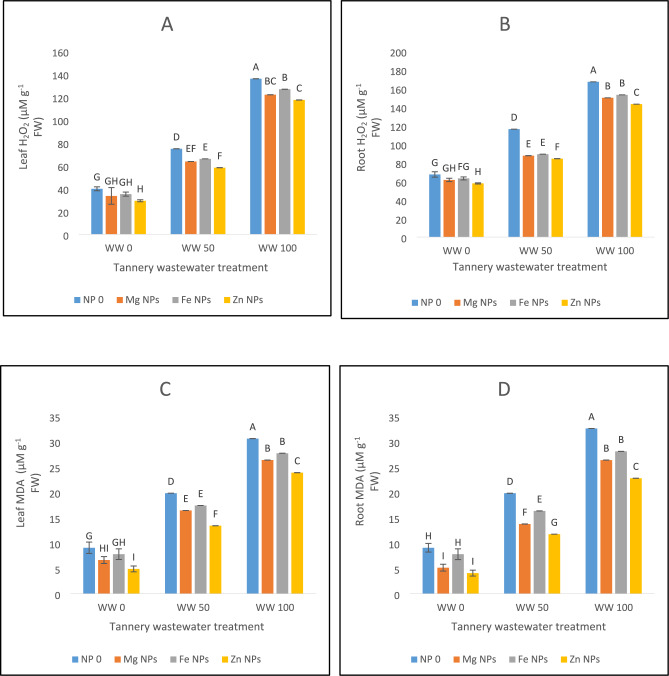

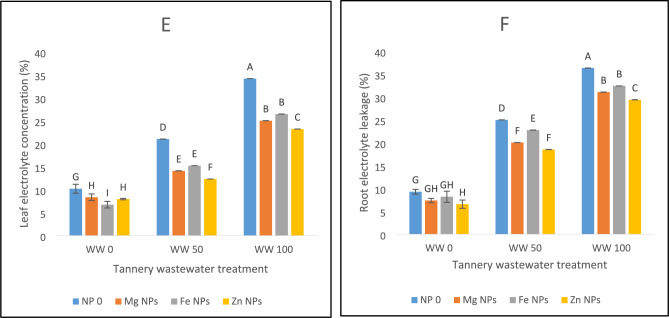
The effects of Fe, Zn and Mg nanoparticles (at concentrations of 25, 50 and 75 mg l^−1^, respectively) on the antioxidant enzyme activity of spinach plants when irrigated with (0%, 50%, and 100%) wastewater from tannery: (**A**) Leaf H_2_O_2_, (**B**) Root H_2_O_2_, (**C**) Leaf MDA, (**D**) Root MDA, (**E**) Leaf Electrolyte leakage, (**F**) Root Electrolyte leakage. Each treatment has three replicates. Letters assigned to the values indicate significance at α = 5%.

Significant increases in H_2_O_2_ levels were seen under wastewater stress in both leaf and root (Fig. 3A,B). Wastewater application of 100% produced the most oxidative stress conditions which was successfully lowered by all the nanoparticle treatments in both leaf and root. Following a 6% lowered production of H_2_O_2_ under wastewater 100% coupled with Fe NPs and a 10% lessened production of H_2_O_2_ under wastewater 100% coupled with Mg NPs treatments, Zn NPs treatment diminished H_2_O_2_ synthesis in leaves by 13% under similar wastewater treatment conditions (Fig. 3A). Roots also showed a similar pattern. A drop of 14% in synthesis of H_2_O_2_ under wastewater 100% coupled with Zn NPs treatment was followed by wastewater 100% combined with Mg NPs treatment (10%) and wastewater 100% coupled with Fe NPs (8%). Wastewater 50% coupled with Zn NPs treatment had the greatest decline in H_2_O_2_ in both leaves (22%) and roots (27%) at 50% wastewater stress level. A relief to the plants in terms of H_2_O_2_ synthesis under wastewater 50% coupled with Mg treatment (14% in leaves, 24% in roots) and wastewater 50% coupled with Fe NPs treatment (12% in leaves, 23% in roots) was noticed (Fig. 3A,B).

Under wastewater stress, MDA levels rose, and 100% wastewater without nanoparticles showed the worst oxidative damage (Fig. 3C,D). However, treatments with nanoparticles significantly lowered MDA levels, demonstrating their protective function. Under extreme stress of wastewater 100%, zinc nanoparticles were the most effective, lowering MDA in roots by 30% and in leaves by 22%. Notable and significant reductions were also produced using iron and magnesium nanoparticles. Zinc nanoparticles exhibited the greatest reduction in MDA levels under 50% wastewater stress, followed by iron and magnesium which were at par with each other. These results highlight the potential of nanoparticles, especially zinc, in reducing oxidative damage brought on by exposure to wastewater (Fig. 3C,D). Electrolyte leakage increased due to wastewater stress in our studies (Fig. 3E,F). Leakage was reduced to 67% in wastewater 100% irrigation with the most efficient zinc concentration, whereas, under extreme stress of 100% wastewater, magnesium nanoparticles also significantly reduced the electrolyte leakage, lowering it to 73% (Fig. 3E,F).

### Antioxidant enzyme activity

**Fig. 4:**
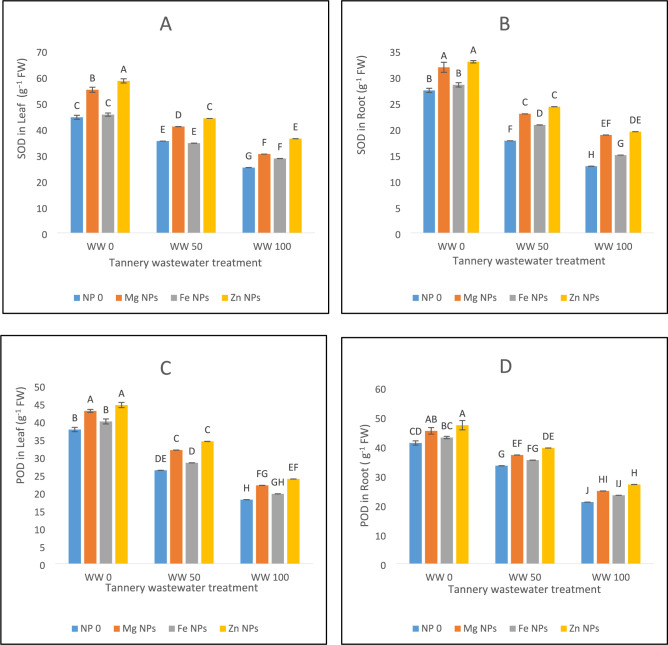

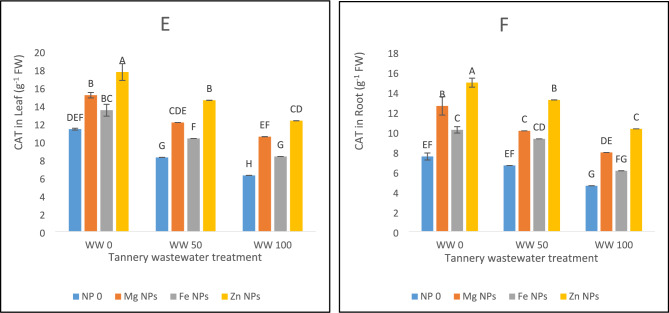
The effects of Fe, Zn and Mg nanoparticles (at concentrations of 25, 50 and 75 mg l^−1^, respectively) on the antioxidant enzyme activity of spinach plants when irrigated with (0%, 50%, and 100%) wastewater from tannery: (**A**) SOD in leaves, (**B**) SOD in roots, (**C**) POD in leaves, (**D**) POD in root, (**E**) CAT in Leaf, (**F**) CAT in roots. Each treatment has three replicates. Letters assigned to the values indicate significance at α = 5%.

The results of current study expressed decreased SOD activity in spinach leaves at both the stress levels of 50 and 100% wastewater by 20% and 43%, accordingly, as compared to control treatment (Fig. 4A). Further, under various circumstances of wastewater treatment, SOD activity was successfully upregulated by nanoparticle (NP) applications. SOD activity was enhanced by 23% under control conditions. Mg NPs application increased SOD by 16% at wastewater 50% compared with control. The greatest improvement of SOD activity at this level was shown by Zn NPs. Mg, Fe, and Zn all increased SOD activity in leaves by 20%, 14%, and 25%, respectively, under severe stress conditions (wastewater 100%) (Fig. 4A). This indicates that zinc is the most effective NP for alleviating oxidative stress by tannery wastewater. Similarly, SOD activity was decreased in root upon exposure to wastewater treatments i.e. by 35% at wastewater 50% and by 58% at wastewater 100% (Fig. 4B). The most effective of the nanoparticles was zinc, which increased the SOD activity by 38% in wastewater 100% and 22% in wastewater 50% in roots. Additionally, magnesium increased SOD activity by 29% in WW100 and 25% in wastewater 50%. Fe improved SOD activity by 16% in wastewater 100% and 19% in wastewater 50%. Zn NPs provide the best protection overall, particularly when wastewater stress levels were high (Fig. 4B).

Wastewater stress decreased POD activity in both leaves and roots (Fig. 4C, D). For leaves, there was 30% decrease at wastewater 50% and 52% at wastewater 100% under control conditions (Fig. 4C), whereas, respective decline of POD for roots was 18% and 49%. Zinc was the most effective nanoparticle at raising POD activity which was 18% high at wastewater 50% and 28% at wastewater 100% for roots, and 31% in wastewater 50% and 32% in wastewater 100% for leaves. Further, magnesium increased POD activity, increasing under wastewater 50% and wastewater 100% for leaves by 21% and 22%, respectively, and under wastewater 100% for roots by 18%. The least effective was Fe, which only improved POD activity in leaves by 8% in wastewater 50% and 9% in wastewater 100% compared with control (Fig. 4C,D).

Similar downfall in CAT activity was obtained in both leaves and roots due to wastewater stress (Fig. 4E,F). It lowered by 45% for leaves and 39% for roots at wastewater 100% in comparison with control, however, Zn NPs improved CAT activity in both leaves and roots under wastewater stress environment compared to control. Likewise, Mg and Fe NPs significantly enhanced CAT activity in both the leaf and root under all the wastewater stresses. Overall, especially under 100% wastewater circumstances, Zn NPs offered the best defense mechanism against oxidative stress (Fig. 4E,F).

### Accumulation of Cr

**Fig. 5:**
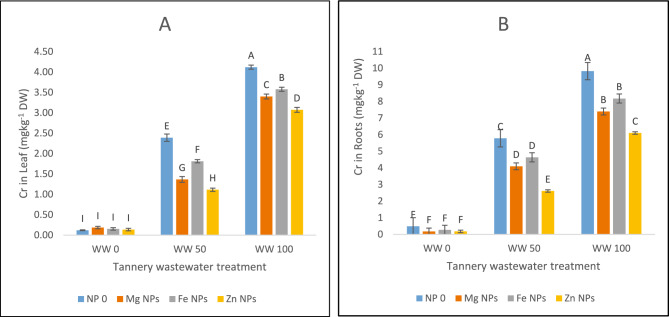
The effects of Fe, Zn and Mg nanoparticles (at concentrations of 25, 50 and 75 mg l^−1^, respectively) on Chromium uptake when irrigated with (0%, 50%, and 100%) wastewater from tannery: (**A**) Chromium in leaves, (**B**) Chromium in roots. Each treatment has three replicates. Letters assigned to the values indicate significance at α = 5%.

Cr was the most prevalent heavy metal in the tannery effluent followed by Pb and Cd (Table 2). Under wastewater stressful conditions, there was a rise in Cr accumulation in both shoots and roots and the greatest amounts were found in case of wastewater 100% treatment under control conditions (4 mg/kg in shoots and 9 mg/kg in roots) (Fig. 5A,B). Zn NPs, when applied, were the most useful in curbing Cr acquisition by the plants. At wastewater 100%, Zn decreased Cr uptake by 25%, Mg by 17% and Fe by 13% in shoots (Fig. 5A). Likewise, under wastewater 100% and 50% stress conditions, Zn NPs reduced absorption of Cr by 38% and 47%, Mg by 25% and 32%, and Fe by 37% and 29%, respectively, by roots (Fig. 5B). Zn NPs remained the most effective in controlling the assimilation of Cr heavy metal by the plants irrigated with tannery wastewater.

### Nutrient accumulation

**Fig. 6:**
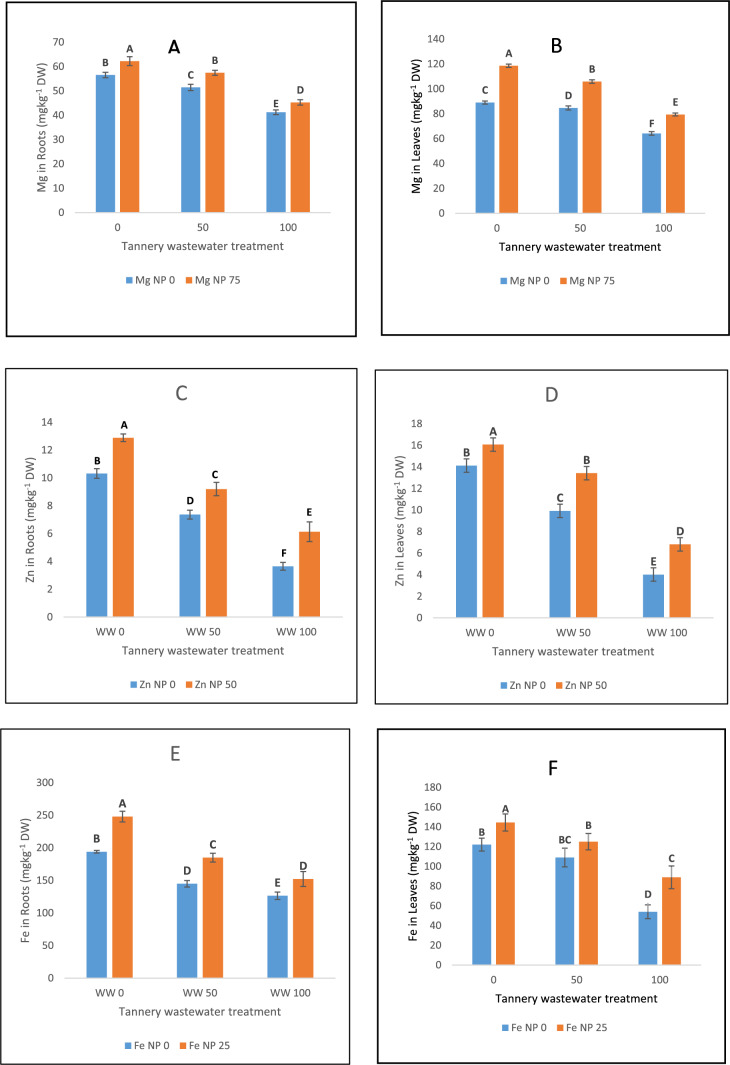
The effects of Fe, Zn and Mg nanoparticles (at concentrations of 25, 50 and 75 mg l^−1^, respectively) on Chromium uptake when irrigated with (0%, 50%, and 100%) wastewater from tannery: (**A**) Mg concentration in roots, (**B**) Mg concentration in leaves, (**C**) Zn concentration in roots, (**D**) Zn concentration in leaves, (**E**) Fe concentration in roots, (**F**) Fe concentration in leaves. Each treatment has three replicates. Letters assigned to the values indicate significance at α = 5%.

In spinach leaves and roots, chromium stress from tannery effluent significantly decreased the uptake of vital minerals including magnesium, zinc and iron (Fig. 6). However, significant improvements in nutritional levels of these minerals were recorded with the use of Zn, Fe, and Mg oxide nanoparticles under both the levels of tannery effluent stress i.e. wastewater 100% and 50%. Under all stress settings, Mg and Fe nanoparticles enhanced magnesium and iron uptake, respectively, while Zn-NPs were more successful in boosting zinc accumulation (Fig. 6).

## Discussion

The study aimed to investigate the effect of tannery wastewater on the morphological, physiological, and biochemical characteristics of *Spinacia oleracea,* and role of FeO, ZnO, and MgO nanoparticles to mitigate heavy metal stress induced by the tannery wastewater. The results highlighted that morphological, physiological, and biochemical characteristics of *Spinacia oleracea* have been improved after the foliar application of FeO, ZnO, and MgO nanoparticles. Reduced plant growth and development are observed in plants growing in soils contaminated with tannery effluent which in turn decreases plant productivity^35^. Tannery effluent carried excess metals are extremely harmful to plant growth and development^36,37^. Spinach, being a sensitive crop, shows reduced overall growth, and root and shoot development when exposed to elevated Cr levels^38,39^ and other heavy metals^2^ in tannery effluent. Tannery wastewater carrying excess metals reduce chlorophyll and carotenoids content, and hinders photosynthetic ability that subsequently lowers plant growth and productivity^40–42^. In our study, under heavy metal toxic conditions, chlorophyll concentration, transpiration rate, stomatal conductance, net photosynthetic rate, water use efficiency and carotenoid contents were increased in plants supplied with combined foliar application of MgO, FeO and ZnO nanoparticles as compared to plants without application of nanoparticles. All these properties are enzymatically controlled and get hampered due to binding of heavy metals with enzymes (proteins) causing structural damages to enzymes, which in turn causes malfunctioning of photosynthetic apparatus and reduces plant growth^39^.

Our findings are supported by reported literature as well. In Indian mustard (*Brassica juncea*), the administration of FeO-NPs enhanced overall plant growth and photosynthetic pigments^43^. In IRRI-6 and Kissan Basmati, FeO-NPs efficiently increased chlorophyll content and antioxidant enzyme activities while promoting biomass accumulation^44^. Application of ZnO NPs resulted in increased length and height of tomato roots and shoots^45^. ZnO NPs used topically at the ideal concentration increased lettuce dry-weight yield by 6.2%^46^. Application of MgO nanoparticles at lower concentrations substantially enhanced the germination of seeds, plant growth (15–22%), biomass (11–19%), along with photosynthetic pigments in *Brassica napus*^11^. Application of 50 mg l^−1^ of MgO and 100 mg l^−1^ of CaCO₃ nanoparticles significantly improved biomass yield, plant growth, and nodulation in groundnut plants^47^. *Zea mays* L. overall growth, development and yield were enhanced by green produced zinc oxide nanoparticles (ZnO-NPs) under cadmium (Cd) stress. Improved mineral nutrient uptake by plants is known to have been involved in reducing absorption of Cr and showing a positive impact on plant metabolic equipment functioning and growth^29^.

In this study, the application of nanoparticles (MgO, FeO and ZnO) resulted in increased concentration of the minerals in plant body. Resultantly, concentrations of H_2_O_2_ and MDA in plants facing stress was decreased due to lowered Cr intake and its toxicity. Though synthesis of H_2_O_2_ is necessary for many of the plant functions but excess of it during stressful situations damages vital functions of the plant^48^. Increased electrolyte leakage and build-up of Cr in plant was noticed in our investigation under tannery effluent treatments. Likewise, antioxidants (SOD, POD and CAT) biosynthesis was suppressed as a result of heavy metal toxicity under tannery effluent irrigation in our results. This damage was repaired significantly upon application of NPs.

Zinc oxide nanomaterials have the capability to lessen levels of reactive oxygen species in plants^49^. Applying ZnO to wheat plants greatly decreased oxidative damage by reducing electrolyte leakage and increased superoxide dismutase and peroxidase activity in the leaves of wheat under Cd stress^50^. By lowering lipid peroxidation and hydrogen peroxide levels, and increasing antioxidant enzyme activities under heavy metal contamination, ZnO-NPs efficiently lowered oxidative stress in rice seedlings^51^. ZnO NPs were applied to wheat which decreased the rate of electrolyte leakage (EL) and raised the activity of antioxidant enzymes in the leaves^52^. In a study by Bidi et al.,^53^ Fe NPs strengthened antioxidant enzyme activity, which decreased electrolyte leakage (EL) and shielded plant cells from oxidative damage, hence reducing oxidative stress in rice plants exposed to arsenic (As) toxicity. In wheat seedlings exposed to heavy metals, magnetic (Fe_3_O_4_) nanoparticles (2000 mg l^−1^), decreased oxidative stress, growth inhibition and MDA production while increasing SOD and POD activities^54^. Applying MgO-NPs to soybean plants decreased their absorption of arsenic, which lowered oxidative stress^28^.

Foliar application of metal based nanoparticles reduced uptake of Cr by plants and improved the contents of Mg, Fe and Zn in roots and shoots of *Spinacia oleracea*. Fe, Mg and Zn can interfere with the uptake of Cr by the plant roots, being ionically similar. Further, foliar application of nanoparticles is more effective in improving internal concentration of minerals in plant than soil application^29,55^. ZnO NPs applied topically at 25, 50, and 100 mg l^−1^ considerably decreased the accumulation of Cr in rice roots and shoots and increased Zn uptake^55^. In red radish plants, foliar application of ZnO and FeO nanoparticles greatly enhanced growth metrics and raised the plants’ Zn and Fe contents^56^. Applying Fe NPs increased the micronutrients’ bioavailability to the plant, which in turn decreased the uptake and accumulation of Cr in the plants^32,57^. Although direct studies on MgO-NPs reducing Cr accumulation are limited, however, their capacity to enhance nutrient consumption and trigger plant defense mechanisms suggests a potential approach to lowering heavy metal toxicity. Mg oxide spray in nano and conventional form at 40 mg l^− 1^ improved magnesium absorption in mint plants^58^. According to our results, using MgO, ZnO, and FeO nanoparticles together may be an effective way to lower heavy metal toxicity and enhance spinach’s (*Spinacia oleracea*) performance.

## Conclusion

The wastewater stress caused serious damage to spinach, leading to hindered plant growth and development. Cr stress alongside other heavy metals, significantly reduced the biomass, impaired photosynthetic capacity, enhanced oxidative stress, upregulated build-up of Cr in plant body and lowered the assimilation of mineral nutrients i.e. Mg, Fe and Zn. Applying metal oxide nanoparticles topically, particularly iron (Fe), magnesium (Mg) and zinc (Zn) NPs, have shown promise in reducing these adverse effects. These nanoparticles decrease Cr absorption and translocation within the plant system in addition to promoting plant growth. Additionally, by increasing the activity of oxidative enzymes, they boost plant’s antioxidant defense mechanisms, increase chlorophyll concentration and enhance photosynthetic efficiency of the plants. Thus, applying Fe, Mg, and Zn nanoparticles topically to the leaves may be a good way to reduce manage the toxicity in spinach, encouraging better growth and increased resistance to heavy metal stress.

## Methods

This research project was conducted in the University of Okara Botanical Garden, using the Departmental labs to examine various factors. The investigation was carried out using a container-based system having a capacity of 5 L in which 4 kg of soil were added. The soil was obtained from the Botanical Garden of University of Okara, Okara. It was obtained at an index of 0–20 cm, with the aid of a stainless steel augar. The obtained soil was autoclaved at 121 °C for 15 min prior to further experimental usage in order to avoid any soil borne microbial impact. Determination of various properties of soil including physical^59^, chemical^60^, and elemental analyses^61^ were performed and details are reported in Tables 1 and 2. The seeds of spinach were acquired from Ayub Research Institute Faisalabad, Pakistan. Before planting, healthier seeds of equal size were separated and germination percentage was recorded. All the seeds were of the same size and were thoroughly checked to remove unhealthy ones. Before planting in pots, the seeds went through surface sterilization using 3% H_2_O_2_ solution by dipping the seeds in the solution for 3 min. The seeds were then thoroughly washed five times with distilled water. Each pot was then meticulously planted with fifteen seeds, ensuring a robust and uniform distribution for subsequent growth and development. In each pot healthy seeds were planted once at a time below 1 cm of upper surface of pot soil. Pot seedlings received irrigation every 24 h, regularly with showers. Seeds thrived in soil and the germination rate was 100%. The tannery wastewater was obtained from the tanning industry in Sialkot and characterized using standard procedures^62^. Plants were irrigated with wastewater at three different concentrations (0%, 50%, and 100%) prepared by diluting the raw tannery water and applied to each pot at 7-day intervals after 15 days of germination. Fe, Zn, Mg nanoparticles were synthesized utilizing the co-precipitation technique^43^. The administration of Fe, Zn, Mg nanoparticles at a concentration of 25, 50 and 75 mg l^− 1^, respectively, was conducted topically on the plants. The initial foliar application of the nanoparticles occurred fifteen days post germination, with the subsequent three applications being spaced one week apart. There were three independent replicates.

**Table 1:** Properties of soil used for experimentation.

Parameters	Units	Value
Sand	%	18
Silt	%	43
Clay	%	39
Organic matter	%	0.72
pH	–	7.6
EC	mS cm^−1^	3.94
Cation exchange capacity	cmol_c_ kg^−1^	5.12
Fe	mg kg^−1^	0.62
Zn	mg kg^−1^	0.47
Mg	mg kg^−1^	0.53

**Table 2:** Characterization of tannery wastewater.

Parameter	Unit	Value
EC	mS cm^−1^	14.39
pH		7.52
COD	mg l^−1^	751.30
BOD5	mg l^−1^	367
Cr (VI)	mg l^−1^	4.04
Cd	mg l^−1^	0.07
Pb	mg l^−1^	1.07
Fe	mg l^−1^	0.57
Zn	mg l^−1^	1.83
Mg	mg l^−1^	1.12

### Harvesting

Plants were sampled and cleaned with distilled water following a 70-day treatment period. Morphological parameters were then examined and recorded. The plants were oven-dried at 70 °C for various analyses.

### Observations and analysis/parameters

Data from *Spinacia oleracea* experimental set were analysed for various physio-biochemical analyses at predetermined intervals, the 15th day of treatment. Seed germination was regularly monitored throughout the third day of the sowing. From twentieth day onwards, morphological measurements were made every day.

### Plant growth and biomass evaluation

The shoot length (i.e., plant height) was measured in centimetres per meter rod from the soil surface to the top of the last node after harvesting and the mean was calculated.

Plants were harvested. The roots were washed and dried with tissue paper. A steel meter rod was used to measure the root’s length from the hypocotyl to the end of root.

The plants were uprooted, washed and dried with tissue paper. Fresh biomass of shoots and roots was measured using an electronic balance. After taking the fresh weight, samples were stored in paper bags in a dry oven at 70 °C till constant weight. The dry biomass from the shoots and roots was measured using an electronic balance.

### Chlorophyll content and photosynthetic parameters

The chlorophyll content of the leaf was assessed through the utilization of spectrophotometer. Fresh foliage samples were procured from the plant to determine the chlorophyll content. A total of six fully developed and vigorously green leaves were systematically selected from each experimental group for the purpose of assessing their chlorophyll concentrations. These selected leaves were then carefully enclosed in labelled plastic bags, preserved in coolers filled with ice, and promptly transported to the laboratory for chlorophyll examination. Chlorophyll contents were then measured^63^. On days characterized by clear skies, the utilization of an infrared gas analyzer (IRGA), Plant Photosynthesis Meter, model 3051C, Zhejiang TOP Cloud-agri Technology Co. Limited, China, was employed to assess variables including gas exchange rates, efficiency of photosynthesis, transpiration rate, and the overall effectiveness of water utilization.

### Measurement of oxidative stress markers and antioxidant enzymes activities

After a period of eight weeks after the sowing process, the measurements were taken to determine electrolyte leakage, malondialdehyde, hydrogen peroxide, as well as antioxidant enzyme activity. In this technique leaf samples were painstakingly eliminated and set then in the cylinders loaded up with a particular measure of distilled water. The samples were autoclaved at 32 °C for 2 h, and during that time the underlying electrical conductivity (EC1) was estimated. From that point onward, the examples were autoclaved at 121 °C for twenty minutes, and the last electrical conductivity (EC2) was estimated. The electrolyte spillage (EL) was determined utilizing the recipe^64^, which gives:

$${\mathrm{EL}} = \, \left( {{\mathrm{EC1}}/{\mathrm{EC2}}} \right){1}00$$

MDA and H_2_O_2_ exercises were assessed following two months of planting. To gauge the malondialdehyde (MDA) levels, 0.25 mg of leaf and root tests were blended in with 5 mL of a 0.1% thiobarbituric corrosive arrangement^65^. The samples were then homogenized prior to being centrifuged at 6000 rpm for 25 min. After centrifugation, 20% H_2_SO_4_ was added, trailed by an additional 15-min centrifugation at 6000 × g. Absorbance readings at 410 nm were utilized to decide hydrogen peroxide content, which was then determined utilizing termination coefficients. To assess antioxidant enzyme action, plant tests were homogenized with 0.5 M phosphate cradle and centrifuged at 12,000 × for around 10 min. The APX activity was determined using the methodologies^66^. Activities of Peroxidase (POD) along with the activities of superoxide dismutase (SOD) in leaves as well in roots were measured with the help of spectrophotometer^67^. Following that, catalase activity (CAT) was also measured^68^.

### Metal-analysis of plants by acid digestion method

For digestion the plants were divided into shoots and roots after harvesting the plants for measurement of fresh biomass. Following this by using an electric oven with a set temperature of 80 °C the samples were dried for 24 h duration. To check for chromium uptake by the plants, a digestion mixture was prepared.

### Determination of heavy metals

The concentration of heavy metal i.e. Chromium (Cr), in the prepared samples was determined by using Atomic Absorption Spectrophotometer (Hitachi Polarized Zeeman AAS, Z-8200, Japan) following the standard procedures^69^.

### Determination of elements (Mg, Fe and Zn)

Elemental analysis of magnesium, iron and zinc in plant shoot and root was performed following the method characterized by Masson et al^70^.

### Statistical analysis

The experiment was arranged in factorial completely randomized design. The data collected were analysed statistically using analysis of variance (ANOVA) for significant differences among the means. For this purpose, SPSS 16.0 software was employed. Treatment means were separated following Least Significance Difference (LSD) test at a significance level of 5% (P < 0.05).

## Data Availability

The dataset can be available from the corresponding author on reasonable request.
